# *RNF213* p.R4810K (c.14429G > A) Variant Determines Anatomical Variations of the Circle of Willis in Cerebrovascular Disease

**DOI:** 10.3389/fnagi.2021.681743

**Published:** 2021-07-15

**Authors:** Futoshi Eto, Takeshi Yoshimoto, Shuhei Okazaki, Kunihiro Nishimura, Shiori Ogura, Eriko Yamaguchi, Kazuki Fukuma, Satoshi Saito, Kazuo Washida, Masatoshi Koga, Kazunori Toyoda, Takaaki Morimoto, Hirofumi Maruyama, Akio Koizumi, Masafumi Ihara

**Affiliations:** ^1^Department of Neurology, National Cerebral and Cardiovascular Center, Suita, Japan; ^2^Department of Cerebrovascular Medicine, National Cerebral and Cardiovascular Center, Suita, Japan; ^3^Department of Neurology, Osaka University Graduate School of Medicine, Suita, Japan; ^4^Department of Preventive Medicine and Epidemiologic Informatics, National Cerebral and Cardiovascular Center, Suita, Japan; ^5^Department of Neurosurgery, Hyogo Prefectural Amagasaki General Medical Center, Amagasaki, Japan; ^6^Department of Health and Environmental Sciences, Graduate School of Medicine, Kyoto University, Kyoto, Japan; ^7^Department of Clinical Neuroscience and Therapeutics, Hiroshima University, Hiroshima, Japan; ^8^Social Health Medicine Welfare Laboratory, Public Interest Incorporated Association Kyoto Hokenkai, Kyoto, Japan

**Keywords:** *RNF213* p.R4810K, the circle of Willis, cerebral circulation, magnetic resonance angiography, single nucleotide polymorphism

## Abstract

**Introduction:**

Dysregulation of the RING finger protein 213 *(RNF213)* gene impairs vascular formation in experimental animal models. In addition, vascular abnormalities in the circle of Willis are associated with cerebrovascular disease. Here, we evaluated the relationship between the East Asian founder variant *RNF213* p.R4810K and consequent anatomical variations in the circle of Willis in cerebrovascular disease.

**Patients and Methods:**

The present study is an observational cross-sectional study. It included patients with acute anterior circulation non-cardioembolic stroke admitted to our institution within 7 days of symptom onset or last-known-well from 2011 to 2019, and those who participated in the National Cerebral and Cardiovascular Center Biobank. We compared anatomical variations of the vessels constituting the circle of Willis between *RNF213* p.R4810K (c.14429G > A) variant carriers and non-carriers using magnetic resonance angiography and assessed the association between the variants and the presence of the vessels constituting the circle of Willis. Patients with moyamoya disease were excluded.

**Results:**

Four hundred eighty-one patients [146 women (30%); median age 70 years; median baseline National Institutes of Health Stroke Scale score 5] were analyzed. The *RNF213* p.R4810K variant carriers (*n* = 25) were more likely to have both posterior communicating arteries (PComAs) than the variant non-carriers (*n* = 456) (56% vs. 13%, *P* < 0.01). Furthermore, variant carriers were less likely to have an anterior communicating artery (AComA) than non-carriers (68% vs. 84%, *P* = 0.04). In a multivariate logistic regression analysis, the association of *RNF213* p.R4810K variant carriers with the presence of both PComAs and the absence of AComA remained significant.

**Conclusion:**

Our findings suggest that the *RNF213* p.R4810K variant is an important factor in determining anatomical variations in the circle of Willis.

## Introduction

RING finger protein 213 (*RNF213*) is a susceptibility gene for large artery atherosclerosis (LAA) (Okazaki et al., 2019) as well as moyamoya disease (MMD), which is a progressive steno-occlusive disease of the circle of Willis (Kamada et al., 2011; Liu et al., 2011). This gene encodes a large protein containing two AAA + ATPases and an E3 ligase domain (Liu et al., 2011; Morito et al., 2014), and plays an important role in regulating vascular endothelial function and angiogenesis (Koizumi et al., 2016). Several studies have revealed that MMD patients in East Asia commonly possess the *RNF213* p.R4810K (c.14429G > A) variant (Koizumi et al., 2016). Notably, in Japan, about 90% of MMD patients have this variant, compared with just 2–3% of the general population (Koizumi et al., 2013; Cao et al., 2016). Approximately 20–25% of Japanese patients with intracranial proximal arterial stenosis, but who do not meet the diagnostic criteria for MMD, also have this variant (Miyawaki et al., 2012).

Genetic variants have major effects on the development of cerebral vasculature, as demonstrated in both clinical and experimental studies. Different genetic backgrounds in mice significantly affect flow-mediated outward remodeling in the bilateral posterior communicating arteries (PComAs) after unilateral occlusion of the middle cerebral artery (MCA) (Faber et al., 2019). Moreover, knockdown of *RNF213* in zebrafish impairs the formation of the cerebral vasculature. A recent clinical study has also reported that the *RNF213* p.R4810K variant is associated with intracranial major artery stenosis or occlusion in anterior cerebral circulation (Matsuda et al., 2017).

We therefore sought to evaluate the relationship between the East Asian founder variant *RNF213* p.R4810K, associated with a single nucleotide polymorphism (SNP; rs112735431), and consequent anatomical variations in the circle of Willis in cerebrovascular disease.

## Materials and Methods

### Study Design and Patients

This observational cross-sectional study was performed at the National Cerebral and Cardiovascular Center (NCVC), Osaka, Japan. The study was conducted in accordance with the Declaration of Helsinki and was approved by the local ethics committees at the NCVC Biobank and Kyoto University. The study was also approved by the Institutional Review Board of NCVC (approval numbers M29-003-9 and M30-145-5). All participants signed a comprehensive consent form at the NCVC.

All patients with ischemic stroke or transient ischemic attack (TIA) who were admitted to our institute within 7 days of symptom onset or last-known-well were prospectively registered to the NCVC Stroke Registry (Yoshimoto et al., 2021). Data for the period from January 2011 to July 2019 were retrospectively reviewed, and patients who met the following criteria were included: (1) admitted with acute anterior circulation non-cardioembolic stroke or TIA; (2) participated in the NCVC Biobank; and, (3) had magnetic resonance imaging (MRI) data available. Patients with MMD were excluded based on diagnostic criteria (Research Committee on the Pathology, and Treatment of Spontaneous Occlusion of the Circle of Willis, and Health Labour Sciences Research Grant for Research on Measures for Intractable Diseases, 2012).

### Genotype Analysis

Peripheral blood samples were obtained from all patients. Genomic DNA was extracted from the buffy coat of blood samples using QIAsymphony SP equipment (Qiagen, Hilden, Germany). Genotyping was performed using TaqMan SNP Assays (Applied Biosystems, Foster City, CA, United States) as described previously (Liu et al., 2011). From the *RNF213* p.R4810K variant genotype analysis, individuals with the GA variant for p.R4810K or the homozygote AA genotype were defined as variant carriers, while individuals with the wild-type homozygote were defined as variant non-carriers.

### Imaging Protocol

MRI was performed using a 3-tesla system (MAGNETOM Verio or Spectra, Siemens, Erlangen, Germany) at admission. Circle of Willis morphology was evaluated by magnetic resonance angiography (MRA) with maximum intensity protection, a method used to reconfigure and prevent overvaluation and overestimation.

**FIGURE 1:**
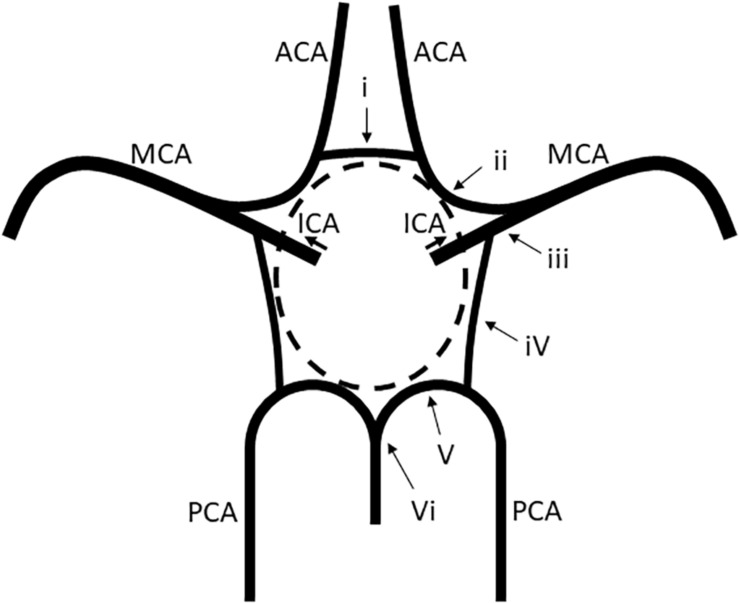
Vessels constituting the circle of Willis. (i) The AComA, (ii) the A1 segment of the ACAs, (iii) the intracranial ICA beyond the PComA bifurcation, (iv) the PComAs, (v) the P1 segment of the PCAs, and (vi) the top of the BA. ACAs, anterior cerebral arteries; AComA, anterior communicating artery; BA, basilar artery; ICAs, internal carotid arteries; PCAs, posterior cerebral arteries; PComAs, posterior communicating arteries.

The vessels constituting the circle of Willis were defined as follows: (i) the anterior communicating artery (AComA), (ii) the A1 segments of the anterior cerebral arteries (ACAs), (iii) the intracranial internal carotid arteries (ICAs) beyond the PComA bifurcation, (iv) the PComAs, (v) the P1 segments of the posterior cerebral arteries (PCAs), and (vi) the top of the basilar artery (BA) (Figure 1). Intracranial ICA was defined as the ICA from the PComA bifurcation to the top of the ICA.

**FIGURE 2:**
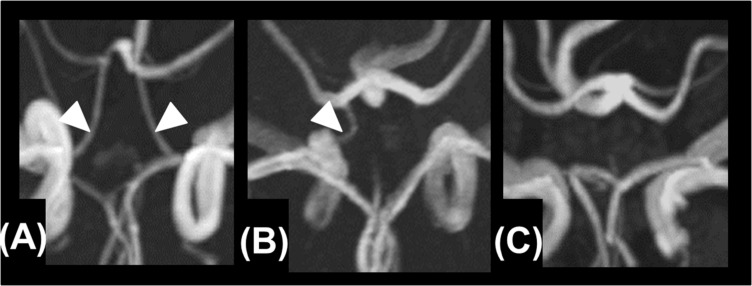
Presence of both vessels **(A)**, unilateral presence of one vessel **(B)**, and bilateral absence of the vessels **(C)**.

Based on the MRA findings, the A1 segments of the ACAs, the intracranial ICAs, the PComAs, and the P1 segments of the PCAs were classified into the presence of both vessels (Figure 2A), a unilateral vessel (Figure 2B), or the absence of both vessels (Figure 2C). The AComA and the top of the BA were classified by the presence and absence of the vessels. The absence of vessels included artery aplasia or hypoplasia (defined as an internal diameter under 1 mm Merkkola et al., 2006). To determine the anatomical variations, the MRA findings of the vessels constituting the circle of Willis and the focal narrowing of the M1 segment of the MCA were independently analyzed by two stroke neurologists (F.E. and T.Y.), who were blind to the clinical information. Joint assessments were carried out for consensus if required.

### Clinical Data Collection

The baseline clinical characteristics were collected from the NCVC Stroke Registry: sex, age, prestroke modified Rankin Scale (mRS) score, medical history (hypertension, diabetes mellitus, dyslipidemia, ischemic heart disease, and chronic kidney disease), atrial fibrillation, current smoking, systolic blood pressure on admission, baseline National Institutes of Health Stroke Scale (NIHSS) score, Alberta Stroke Program Early Computed Tomographic Score (ASPECTS) on diffusion-weighted imaging (DWI) or computed tomography (CT), the presence of vessels constituting the circle of Willis (AComA, both A1 segments of the ACAs, both intracranial ICAs, and both PComAs), completeness of the circle of Willis, focal narrowing of the M1 segment of the MCA, stroke subtype, and clinical outcome (mRS score at discharge and in-hospital mortality). Hypertension was diagnosed at hospital discharge or from the use of antihypertensive medications before the index stroke/TIA. Diabetes mellitus was diagnosed at hospital discharge or from antidiabetic treatment before the index stroke/TIA. Dyslipidemia was diagnosed at hospital discharge or from lipid-lowering therapy before the index stroke/TIA. A complete circle of Willis was defined by the presence of all of the vessels that constitute the circle of Willis. Stroke subtype, including LAA, small vessel occlusion (SVO), and other/undetermined etiology, was determined by stroke neurologists according to the Trial of ORG 10172 in Acute Stroke Treatment criteria (Adams et al., 1993).

### Statistical Analyses

Data are summarized as the median [interquartile range (IQR)] for continuous variables and the frequencies (percentages) for categorical variables. Patients were divided into two groups: *RNF213* p.R4810K variant carriers and non-carriers. The clinical characteristics, presence of vessels constituting the circle of Willis, and clinical outcomes were compared between groups using the Mann–Whitney *U*-test or Fisher’s exact test, as appropriate. We assessed the correlations between anatomical variations of the circle of Willis (both PComAs and AComA) and the *RNF213* p.R4810K variant using the following models. For Model 1, a multivariate logistic regression model was created to investigate the association between anatomical variations of the circle of Willis (both PComAs and AComA) and the *RNF213* p.R4810K variant. Variables with *P* < 0.10 (women, age, focal narrowing of the M1 segment of MCA, and stroke subtype) were selected for the multivariate model. For Model 2, associations between anatomical variations of the circle of Willis (both PComAs and AComA) and the *RNF213* p.R4810K variant were explored using a multivariate logistic regression model; the stepwise method was used for variable selection. To exclude statistically non-significant variables from the logistic regression model, bidirectional stepwise variable selection was used, with a *P*-value for entry of 0.05 and a *P*-value for removal of 0.10. Odds ratios (ORs) with 95% confidence intervals (CIs) were also calculated. We also analyzed the intraclass correlation coefficients for the evaluation of vessels constituting the circle of Willis.

The enrolled patients were then divided into three groups according to stroke subtype (LAA, SVO, and other/undetermined etiology). Baseline characteristics, including the presence of vessels constituting the circle of Willis, were compared. We evaluated the associations between the presence of vessels constituting the circle of Willis and the stroke subtype. All analyses were performed using JMP 14.0.0 statistical software (SAS Institute Inc., Cary, NC, United States).

## Results

**FIGURE 3:**
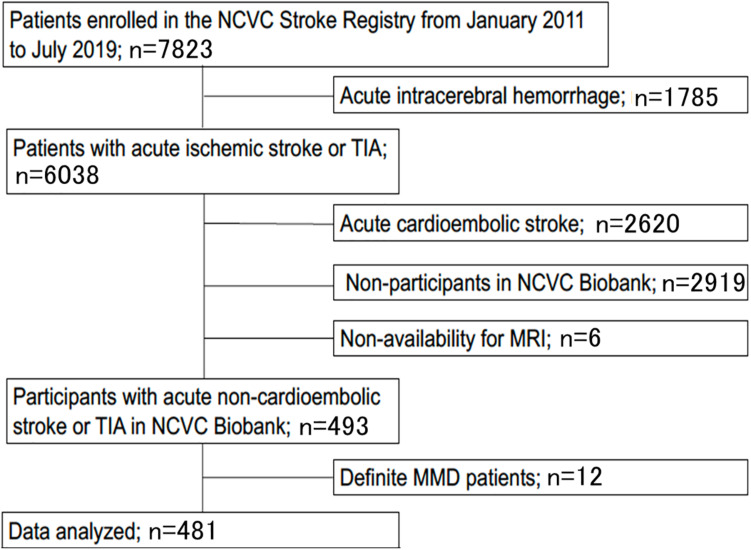
Study flow chart. Patients were excluded based on acute intracerebral hemorrhage (*n* = 1,785), acute cardioembolic stroke (*n* = 2,620), non-participants at the NCVC Biobank (*n* = 2,919), non-availability of MRI (*n* = 6), and MMD cases (*n* = 12). MMD, moyamoya disease; MRI, magnetic resonance imaging; NCVC, National Cerebral, and Cardiovascular Center; TIA, transient ischemic attack.

**FIGURE 4:**
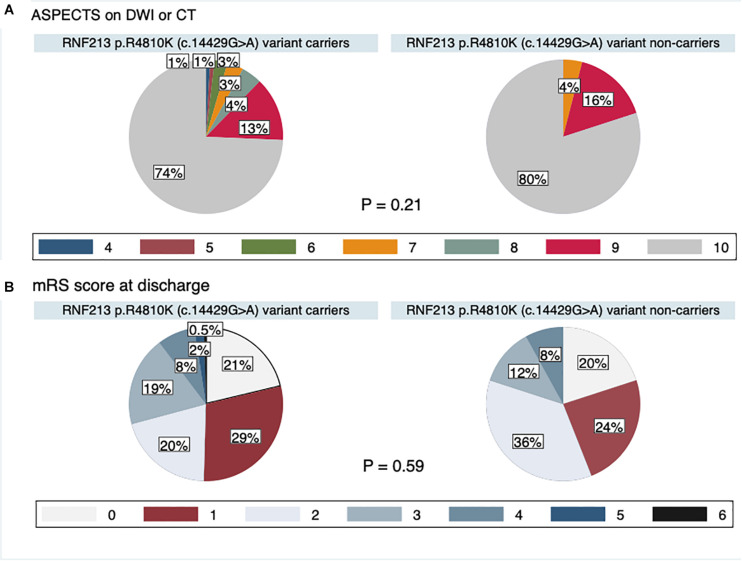
Pie charts of ASPECTS on DWI or CT **(A)** and mRS score at discharge **(B)**.

The study flow chart is provided in Figure 3. From January 2011 to July 2019, 7823 patients were enrolled in the NCVC Stroke Registry. Of these, 481 patients [146 (30%) women, median (IQR) age of 70 (57–78) years] met our study criteria. Patients were excluded because of acute intracerebral hemorrhage (*n* = 1785), acute cardioembolic stroke (*n* = 2620), non-participants at the NCVC Biobank (*n* = 2919), non-availability of MRI (*n* = 6), and MMD cases (*n* = 12). Of the 481 patients [146 women (30%); median (IQR) age 70 (57–78) years, median NIHSS score 5 (2–5)] enrolled in the present study, 25 patients (5.2%) had the GA variant for p.R4810K and no patients had the homozygote AA genotype. The most common comorbidities were hypertension (80%) and dyslipidemia (68%). Forty-nine patients (11%) had a complete circle of Willis. The median (IQR) mRS score at discharge was 1 (1–3) and the in-hospital mortality rate was 0.4%. The distributions of ASPECTS on DWI or CT and mRS scores at discharge in variant carriers and non-carriers are shown in Figure 4.

Compared with *RNF213* p.R4810K (c.14429G > A) non-carriers (*n* = 456), carriers (*n* = 25) were more frequently women (52% vs. 29%, *P* = 0.02), younger (62 years vs. 70 years, *P* < 0.01), had focal narrowing of the M1 segment of the MCA (44% vs. 12%, *P* < 0.01), had a lower presence of AComA (68% vs. 84%, *P* = 0.04), and a higher presence of both PComAs (56% vs. 13%, *P* < 0.01). The baseline characteristics of *RNF213* p.R4810K variant carriers and non-carriers are shown in Table 1. Compared with patients with the unilateral presence or absence of PComAs (*n* = 406), patients with the presence of both PComAs (*n* = 75) were more frequently women (41% vs. 28%, *P* = 0.03), younger (median 62 years vs. 71 years, *P* < 0.01), less hypertensive (69% vs. 82%, *P* = 0.02), had more focal narrowing of the M1 segment of the MCA (26% vs. 12%, *P* = 0.02), and more frequently *RNF213* p.R4810K variant carriers (19% vs. 3%, *P* < 0.01). In the multivariate analysis using the stepwise model (Model 2), patients having both PComAs were more frequently younger (per 1-year increase; OR 0.71, 95% CI 0.59–0.85) and were significantly associated with the variant (OR 8.61, 95% CI 3.48–21.31). The patient baseline characteristics, stratified according to PComA patency state, are shown in Table 2. Two models were used to evaluate the associations between PComAs patency state and the factors shown in Table 3. Compared with patients with AComA (*n* = 401), those without AComA (*n* = 80) were older (median 75 years vs. 69 years, *P* = 0.03), and more frequently the variant carriers (11% vs. 4%, *P* = 0.04). In the multivariate analysis using the stepwise model (Model 2), the absence of AComA were also significantly associated with the variant (OR 2.38, 95% CI 1.02–5.88), as shown in Tables 4, 5. There were no associations between the other vessels constituting the circle of Willis and the *RNF213* p.R4810K variant.

**TABLE 1:** Baseline characteristics between *RNF213* p.R4810K (c.14429G > A) variant carriers and non-carriers.

		*RNF213* p.R4810K (c.14429G > A) variant carriers		
Variables	All (*n* = 481)	Carriers (*n* = 25)	Non-carriers (*n* = 456)	*P*
Women, *n* (%)	146 (30)	13 (52)	133 (29)	0.02
Age, median (IQR), years	70 (57–78)	62 (45–70)	70 (58–79)	<0.01
Prestroke mRS score, median (IQR)	0 (0–0)	0 (0–0)	0 (0–0)	0.57
Baseline systolic BP, median (IQR), mmHg	162 (144–177)	162 (140–175)	161 (150–177)	0.66
**Medical history**				
Hypertension, *n* (%)	384 (80)	20 (80)	364 (80)	1.00
Diabetes mellitus, *n* (%)	135 (28)	8 (32)	127 (28)	0.65
Dyslipidemia, *n* (%)	325 (68)	15 (60)	310 (68)	0.39
Ischemic heart disease, *n* (%)	21 (4)	2 (8)	25 (6)	0.64
Chronic kidney disease, *n* (%)	19 (4)	12 (48)	145 (32)	0.12
Atrial fibrillation, *n* (%)	9 (2)	0 (0)	9 (2)	1.00
Current smoking, *n* (%)	272 (57)	14 (56)	258 (57)	1.00
Baseline NIHSS score, median (IQR)	5 (2–5)	3 (2–6)	5 (2–5)	0.26
ASPECTS on DWI or CT, median (IQR) (*n* = 465)	10 (9–10)	10 (10–10)	10 (9–10)	0.43
**Formation of vessels constituting the circle of Willis**				
AComA, *n* (%)	401 (83)	17 (68)	384 (84)	0.04
Both A1 segments of ACAs, *n* (%)	424 (88)	19 (76)	403 (88)	0.11
Both intracranial ICAs, *n* (%)	467 (97)	23 (92)	444 (97)	0.16
Both PComAs, *n* (%)	75 (16)	14 (56)	61 (13)	<0.01
Both P1 segments of PCAs, *n* (%)	385 (80)	18 (72)	362 (79)	0.45
Top of BA, *n* (%)	478 (99)	24 (96)	455 (99)	0.10
Focal narrowing of the M1 segment of MCA, *n* (%)	66 (14)	11 (44)	55 (12)	<0.01
Complete circle of Willis, *n* (%)	49 (11)	5 (20)	43 (9)	0.09
**Stroke subtype**				<0.01
Large artery atherosclerosis, *n* (%)	139 (29)	14 (56)	125 (27)	
Small vessel occlusion, *n* (%)	135 (28)	5 (20)	130 (29)	
Other/undetermined etiology, *n* (%)	185 (38)	4 (16)	181 (40)	
Transient ischemic attack, *n* (%)	22 (5)	2 (8)	20 (4)	0.32
mRS score at discharge, median (IQR)	1 (1–3)	2 (1–2)	1 (1–3)	0.96
In-hospital mortality, *n* (%)	2 (0.4)	0	2 (0.4)	1.00

*ACAs, anterior cerebral arteries; AComA, anterior communicating artery; ASPECTS, Alberta Stroke Program Early CT Score; BA, basilar artery; BP, blood pressure; CT, computed tomography; DWI, diffusion-weighted imaging; ICAs, internal carotid arteries; IQR, interquartile range; MCA, middle cerebral artery; mRS, modified Rankin Scale; NIHSS, National Institutes of Health Stroke Scale; PCAs, posterior cerebral arteries; PComAs, posterior communicating arteries.*

**TABLE 2:** Baseline characteristics between patients with the presence and absence of both PComAs.

	Both PComAs		
Variables	Presence (*n* = 75)	Absence (*n* = 406)	*P*
Women, *n* (%)	31 (41)	115 (28)	0.03
Age, median (IQR), years	62 (46–73)	71 (60–79)	<0.01
Prestroke mRS score, median (IQR)	0 (0–0)	0 (0–0)	0.72
Baseline systolic BP, median (IQR), mmHg	162 (144–179)	158 (143–182)	0.75
**Medical history**			
Hypertension, *n* (%)	52 (69)	332 (82)	0.02
Diabetes mellitus, *n* (%)	20 (27)	115 (28)	0.89
Dyslipidemia, *n* (%)	46 (61)	279 (69)	0.23
Ischemic heart disease, *n* (%)	3 (4)	24 (6)	0.78
Chronic kidney disease, *n* (%)	12 (48)	145 (32)	0.12
Atrial fibrillation, *n* (%)	8 (2)	1 (1)	1.00
Current smoking, *n* (%)	19 (25)	138 (34)	0.18
Baseline NIHSS score, median (IQR)	5 (1–5)	5 (2–5)	0.33
ASPECTS on DWI or CT, median (IQR) (*n* = 465)	10 (9–10)	10 (10–10)	0.21
**Formation of vessels constituting the circle of Willis**			
AComA, *n* (%)	61 (81)	340 (84)	0.61
Both A1 segments of ACAs, *n* (%)	69 (92)	353 (87)	0.26
Both intracranial ICAs, *n* (%)	74 (99)	393 (97)	0.71
Both P1 segments of PCAs, *n* (%)	63 (84)	317 (78)	0.28
Top of BA, *n* (%)	75 (100)	404 (99)	1.00
Focal narrowing of the M1 segment of MCA, *n* (%)	15 (20)	51 (13)	0.10
**Stroke subtype**			<0.01
Large artery atherosclerosis, *n* (%)	16 (21)	123 (30)	
Small vessel occlusion, *n* (%)	18 (24)	117 (29)	
Other/undetermined etiology, *n* (%)	39 (52)	146 (36)	
Transient ischemic attack, *n* (%)	2 (3)	20 (5)	0.55
**Outcome**			
mRS score at discharge, median (IQR)	1 (0–3)	2 (1–3)	0.59
In-hospital mortality, *n* (%)	0	2 (0.5)	1.00
*RNF213* p.R4810K (c.14429G > A) variant carriers, *n* (%)	14 (19)	11 (3)	<0.01

*ACAs, anterior cerebral arteries; AComA, anterior communicating artery; ASPECTS, Alberta Stroke Program Early CT Score; BA, basilar artery; BP, blood pressure; CT, computed tomography; DWI, diffusion-weighted imaging; ICAs, internal carotid arteries; IQR, interquartile range; MCA, middle cerebral artery; mRS, modified Rankin Scale; NIHSS, National Institutes of Health Stroke Scale; PCAs, posterior cerebral arteries; PComAs, posterior communicating arteries.*

**TABLE 3:** Logistic regression analyses for the presence of both PComAs.

	Unweighted univariable	Model 1	Model 2
	
Variables		OR (95% CI)	
Women	1.78 (1.07–2.96)	1.50 (0.85–2.63)	–
Age (per 10-year increase)	0.67 (0.56–0.79)	0.73 (0.60–0.88)	0.71 (0.59–0.85)
Hypertension	0.50 (0.29–0.87)	0.76 (0.40–1.42)	–
Diabetes mellitus	0.92 (0.53–1.60)		−
Dyslipidemia	0.72 (0.43–1.20)	−	−
Ischemic heart disease	0.66 (0.19–2.26)	−	−
Chronic kidney disease	0.66 (0.38–1.15)	−	−
Atrial fibrillation	0.67 (0.08–5.46)	−	
Current smoking	0.59 (0.36–0.96)	−	−
Focal narrowing of the M1 segment of MCA	1.74 (0.91–3.29)	0.95 (0.43–2.12)	−
Large artery atherosclerosis	0.62 (0.35–1.13)	0.54 (0.26–1.10)	0.50 (0.25–0.99)
*RNF213* p.R4810K (c.14429G > A) variant carriers	8.24 (3.58–18.98)	8.08 (3.17–20.59)	8.61 (3.48–21.31)

*Model 1; adjusted for variables with p < 0.10 (women, age, focal narrowing of the M1 segment of MCA, and large artery atherosclerosis). Model 2; forward–backward stepwise method. CI, confidence interval; MCA, middle cerebral arteries; OR, odds ratio; PComAs, posterior communicating arteries.*

**TABLE 4:** Baseline characteristics between the presence and absence of AComA.

	AComA		
Variables	Absence (*n* = 80)	Presence (*n* = 401)	*P*
Women, *n* (%)	32 (40)	114 (28)	0.05
Age, median (IQR), years	75 (61–82)	69 (57–78)	0.03
Prestroke mRS score, median (IQR)	0 (0–0)	0 (0–0)	0.70
Baseline systolic BP, median (IQR), mmHg	158 (134–170)	162 (144–178)	0.05
**Medical history**			
Hypertension, *n* (%)	66 (83)	318 (79)	0.65
Diabetes mellitus, *n* (%)	16 (20)	119 (30)	0.10
Dyslipidemia, *n* (%)	54 (68)	271 (68)	1.00
Ischemic heart disease, *n* (%)	7 (9)	20 (5)	0.20
Chronic kidney disease, *n* (%)	37 (46)	120 (30)	<0.01
Atrial fibrillation, *n* (%)	4 (5.0%)	5 (1.2%)	0.05
Current smoking, *n* (%)	36 (45)	236 (59)	0.03
Baseline NIHSS score, median (IQR)	3 (2–5)	5 (2–5)	0.21
ASPECTS on DWI or CT, median (IQR) (*n* = 465)	10 (9–10)	10 (9–10)	0.81
**Formation of vessels constituting the circle of Willis**			
Both A1 segments of ACAs, *n* (%)	71 (89)	351 (88)	0.85
Both intracranial ICAs, *n* (%)	78 (98)	389 (97)	1.00
Both PComAs, *n* (%)	14 (18)	61 (15)	0.61
Both P1 segments of PCAs, *n* (%)	62 (78)	318 (79)	0.76
Top of BA, *n* (%)	80 (100)	399 (99)	1.00
Focal narrowing of the M1 segment of MCA, *n* (%)	6 (8)	60 (15)	0.11
**Stroke subtype**			<0.01
Large artery atherosclerosis, *n* (%)	16 (20)	123 (31)	
Small vessel occlusion, *n* (%)	11 (14)	124 (31)	
Other/undetermined etiology, *n* (%)	39 (49)	146 (36)	
Transient ischemic attack, *n* (%)	14 (17.5%)	8 (2.0%)	<0.01
**Outcome**			
mRS score at discharge, median (IQR)	1 (1–3)	2 (1–3)	0.50
In-hospital mortality, *n* (%)	0	2 (0.5)	1.00
*RNF213* p.R4810K (c.14429G > A) variant carriers, *n* (%)	9 (11)	17 (4)	0.04

*ACAs, anterior cerebral arteries; AComA, anterior communicating artery; ASPECTS, Alberta Stroke Program Early CT Score; BA, basilar artery; BP, blood pressure; CT, computed tomography; DWI, diffusion-weighted imaging; ICAs, internal carotid arteries; IQR, interquartile range; MCA, middle cerebral artery; mRS, modified Rankin Scale; NIHSS, National Institutes of Health Stroke Scale; PCAs, posterior cerebral arteries; PComAs, posterior communicating arteries.*

**TABLE 5:** Logistic regression analyses for the absence of AComA.

	Unweighted univariable	Model 1	Model 2
	
Variables		OR (95% CI)	
Women	1.67 (1.02–2.78)	1.18 (0.95–2.17)	−
Age (per 10-year increase)	1.15 (0.97–1.37)	1.10 (0.88–1.37)	−
Baseline systolic BP (per 10 mmHg increase)	0.91 (0.83–1.00)	0.89 (0.81–0.99)	0.91 (0.73–1.01)
Hypertension	2.00 (1.15–3.45)	−	
Diabetes mellitus	0.59 (0.33–1.06)	0.58 (0.31–1.10)	0.59 (0.32–1.09)
Dyslipidemia	1.00 (0.60–1.67)	−	−
Ischemic heart disease	1.82 (0.75–4.55)	−	−
Chronic kidney disease	2.00 (1.23–3.33)	1.49 (0.79–2.78)	1.96 (1.19–3.23)
Atrial fibrillation	4.17 (1.10–16.67)	−	
Current smoking	0.57 (0.35–0.93)	0.70 (0.39–1.25)	0.65 (0.40–1.08)
Focal narrowing of the M1 segment of MCA	0.46 (0.19–1.11)	−	
Large artery atherosclerosis	0.56 (0.31–1.02)	−	
*RNF213* p.R4810K (c.14429G > A) variant carriers	2.56 (1.11–5.88)	2.50 (1.08–6.67)	2.38 (1.02–5.88)

*Model 1; adjusted for variables with p < 0.10 (women, age, baseline systolic BP, diabetes mellitus, chronic kidney disease, and current smoking). Model 2; forward–backward stepwise method. AComA, anterior communicating artery; BP, blood pressure; CI, confidence interval; MCA, middle cerebral artery; OR, odds ratio.*

The intraclass correlation coefficients for the evaluation of vessels constituting the circle of Willis were as follows: 0.97 (95% CI 0.97–0.98) for AComA, 0.98 (95% CI 0.98–0.99) for both A1 segments of the ACAs, 1.00 for both intracranial ICAs, 1.00 for the top of the BA, 0.97 (95% CI 0.97–0.98) for both PComAs, and 0.96 (95% CI 0.95–0.97) for both P1 segments of the PCAs.

There were no significant differences in the vessels constituting the circle of Willis for each stroke subtype, except that the presence of AComA was significantly higher in patients with SVO (LAA 89% vs. SVO 92% vs. other/undetermined etiology 79%, *P* < 0.01). Moreover, the frequency of *RNF213* p.R4810K variant carriers were significantly higher in patients with LAA (LAA 10% vs. SVO 4% vs. other/undetermined etiology 2%, *P* < 0.01). A comparison of the baseline characteristics by stroke subtype is shown in Supplementary Table 1, in the online-only Data Supplement.

## Discussion

The current study provides relevant new data about the associations between vessels constituting the circle of Willis and the *RNF213* p.R4810K variant carrier state. Our results indicate that the East Asian founder variant of *RNF213* affects the vascular formation of the circle of Willis. *RNF213* p.R4810K variant carriers had a higher frequency of the presence of both PComAs and the absence of AComA compared with non-carriers, even after adjusting for differences in baseline characteristics using a stepwise method. These findings reinforce the idea of anterior cerebral circulation failure even in *RNF213* p.R4810K variant carriers who do not satisfy the diagnostic criteria for MMD (Research Committee on the Pathology, and Treatment of Spontaneous Occlusion of the Circle of Willis, and Health Labour Sciences Research Grant for Research on Measures for Intractable Diseases, 2012). To our knowledge, no other SNPs have been reported to be associated with such substantial anatomical variations in the circle of Willis as in the current findings for the *RNF213* p.R4810K variant.

In terms of the variation of vessels constituting the circle of Willis, a previous study evaluated the intracranial arterial morphology of healthy subjects and reported that 21% (110/525) had an absence of AComA, 19% (100/525) had both PComAs, and 20.9% had a complete circle of Willis; however, the *RNF213* p.R4810K variant was not examined in this study (Kondori et al., 2017). In the variant carriers in the present study, the rate of absence of AComA was 32% (8/25), the rate of both PComAs was 56% (14/25), and a complete circle of Willis was present in 20% (5/25) of individuals. Thus, compared with the healthy subjects in the previous report (Kondori et al., 2017), our results showed higher rates of an absence of AComA and the presence of both PComAs in variant carriers. These results suggest that this variant affects anatomical variations in the circle of Willis. In the variant non-carriers, the rate of a complete circle of Willis was very low, at 9% (43/456), and the presence of both PComAs was only 13% (61/453); these findings likely reflect the fact that our study included only patients with a history of ischemic stroke/TIA.

In a previous study examining sex differences in stroke (Arboix et al., 2014), women generally had more stroke events at an older age and a higher number of stroke events than men because of a longer life expectancy. In the present study, although patients with the *RNF213* p.R4810K variant were more frequently women and younger, as previously reported (Kamimura et al., 2019; Okazaki et al., 2019), there were no differences in clinical outcomes, including mRS score at discharge and in-hospital mortality. To clarify the impact of this variant on clinical outcomes, long-term outcomes should be investigated in future research.

Advanced MMD is characterized by the discontinuity or disappearance of PComAs in Suzuki staging (Jin et al., 2011), probably as a result of arteriopathy extending proximally into these vessels. This seems to contradict our finding that variant carriers had increased patency of PComAs. However, this inconsistency may be explained by the different stages of *RNF213*-related vasculopathy between variant carriers with advanced MMD and those with sporadic ischemic stroke or TIA in our cohort (Okazaki et al., 2019; Hosoki et al., 2021), which excluded MMD. That is, patients with advanced MMD, who represent the extreme end of the disease spectrum of *RNF213-*related vasculopathy, have terminal ICA stenosis or occlusion. Anterior circulation insufficiency no longer activates compensatory mechanisms for collateral flow because of the arteriopathy of PComAs. In contrast, patients on the less severe end of the *RNF213-*related vasculopathy spectrum may still have activation of the mechanisms that compensate for insufficient anterior circulation through the PComAs, PCAs, and meningeal arteries (Song et al., 2019). Both the continuity and differences between MMD and non-moyamoya ischemic stroke in the same variant carriers should be further clarified to establish and stratify treatment strategies for *RNF213-*related vasculopathy.

Another explanation of PComA patency may be attributable to the embryonic development of cerebral circulation. In the embryonic stage, the neural crest gives rise to pericytes, thereby contributing to the formation of anterior cerebral circulation, which specifically includes primitive ICAs but not the vertebrobasilar arteries of mesodermal origin (Komiyama, 2017a). Therefore, failures in the differentiation and migration of neural crest cells may lead to the insufficiency of anterior cerebral circulation, including MMD, multisystemic smooth muscle dysfunction syndrome caused by *ACTA2* mutation, and PHACE syndrome, which are collectively named vascular neurocristopathy (cardio-cephalic neural crest syndrome) (Komiyama, 2017b). Thus, anatomical variations of the circle of Willis in variant carriers may not be acquired, but might be primarily determined in the embryonic stage. Additional studies in healthy carriers or other ischemic stroke and TIA cohorts are required to determine precisely when the *RNF213* p.R4810K variant leads to the observed arterial variations. This gene polymorphism may also be involved in the relationship between cerebrovascular pathology and Alzheimer’s disease (AD) because vascular abnormalities affect the accumulation of amyloid protein and the progression of behavioral abnormalities in both AD patients and animal models (Farkas and Luiten, 2001). Another report (Roher et al., 2003) has demonstrated a relationship between circle of Willis atherosclerosis and AD, thus strengthening the importance of cerebrovascular architecture in AD.

There are several limitations in the present study. The first and major limitation was the lack of genetic and neuroimaging data from healthy volunteers, which was the result of ethical and procedural issues. Because only patients with previous ischemic stroke/TIA were enrolled in this study, it will be necessary to evaluate healthy subjects in a future validation cohort. Second, the anatomical variations of intracranial arteries were evaluated by MRA in the current study. Digital subtraction angiography is the gold standard technique and is superior to MRA in the evaluation of cerebrovascular architecture, although MRA has an advantage as a non-invasive imaging modality. Third, only 14% of patients enrolled in the study gave full consent for their data to be collected and stored at the NCVC Biobank; such selection bias may have led to an overestimation of the prevalence of the *RNF213* p.R4810K variant. Fourth, although a previous whole-genome microarray analysis demonstrated associations between *RNF 213* polymorphisms and AD (Bai et al., 2014), cognitive status was not evaluated in the present study because the cerebrovascular disease affects cognitive function, and thus makes the diagnosis of AD difficult in an acute stroke setting. However, the results of the current study may stimulate future exploration of the association between AD and the *RNF 213* p.R4810K variant. Finally, we were unable to entirely rule out acquired changes in intracranial arteries caused by atherosclerosis. However, the association between the presence of both PComAs and the *RNF213* variant remained even after adjusting for baseline factors using the stepwise method.

## Conclusion

*RNF213* p.R4810K variant carriers had higher rates of the presence of both PComAs and an absence of AComA than non-carriers. In variant carriers, an insufficiency of anterior cerebral circulation arteries may be accompanied by compensatory collateral flow through the PComAs, thus affecting the anatomy of the circle of Willis.

## Data Availability Statement

The raw data supporting the conclusions of this article will be made available by the authors, without undue reservation.

## Ethics Statement

The studies involving human participants were reviewed and approved by the National Cerebral and Cardiovascular Center Biobank and Kyoto University. The patients/participants provided their written informed consent to participate in this study.

## Author Contribution

FE wrote the first draft of the manuscript, evaluated MRA findings, performed the statistical analysis, and interpreted the data. TY revised the draft of the manuscript, evaluated the MRA findings, provided advice on the statistical analysis, and interpreted the data. KN provided advice on the statistical analysis. SOk, SOg, EY, KF, SS, KW, MK, KT, and HM critically revised the article for important intellectual content. TM and AK supported the genotyping from peripheral blood samples and critically revised the article for important intellectual content. MI designed the study, interpreted the data, and wrote the final draft of the manuscript. All authors contributed to the article and approved the submitted version.

## Conflict of Interest

The authors declare that the research was conducted in the absence of any commercial or financial relationships that could be construed as a potential conflict of interest.

## References

[B1] Adams H. P. Jr. Bendixen B. H. Kappelle L. J. Biller J. Love B. B. Gordon D. L. (1993). Classification of subtype of acute ischemic stroke. definitions for use in a multi-center clinical trial. TOAST. trial of Org 10172 in acute stroke treatment. *Stroke* 24 35–41. 10.1161/01.str.24.1.35 7678184

[B2] Arboix A. Cartanya A. Lowak M. Garcia-Eroles L. Parra O. Oliveres M. (2014). Gender differences and woman-specific trends in acute stroke: results from a hospital-based registry (1986-2009). *Clin. Neurol. Neurosurg.* 127 19–24. 10.1016/j.clineuro.2014.09.024 25459238

[B3] Bai Z. Stamova B. Xu H. Ander B. P. Wang J. Jickling G. C. (2014). Distinctive RNA expression profiles in blood associated with Alzheimer’s disease after accounting for white matter hyperintensities. *Alzheimer Dis. Assoc. Disord.* 28 226–233. 10.1097/WAD.0000000000000022 24731980 PMC4139468

[B4] Cao Y. Kobayashi H. Morimoto T. Kabata R. Harada K. H. Koizumi A. (2016). Frequency of RNF213 p.R4810K, a susceptibility variant for moyamoya disease, and health characteristics of carriers in the Japanese population. *Environ. Health Prev. Med.* 21 387–390. 10.1007/s12199-016-0549-8 27365075 PMC5305994

[B5] Faber J. E. Zhang H. Rzechorzek W. Dai K. Z. Summers B. T. Blazek C. (2019). Genetic and environmental contributions to variation in the posterior communicating collaterals of the circle of Willis. *Transl. Stroke Res.* 10 189–203. 10.1007/s12975-018-0626-y 29589286

[B6] Farkas E. Luiten P. G. M. (2001). Cerebral microvascular pathology in aging and Alzheimer’s disease. *Prog. Neurobiol.* 64 575–611. 10.1016/s0301-0082(00)00068-x 11311463

[B7] Hosoki S. Yoshimoto T. Ihara M. (2021). A case of hemichorea in RNF213-related vasculopathy. *BMC Neurol.* 21:32. 10.1186/s12883-021-02061-2067 PMC7821645 33482763

[B8] Jin Q. Noguchi T. Irie H. Kawashima M. Nishihara M. Takase Y. (2011). Assessment of moyamoya disease with 3.0-T magnetic resonance angiography and magnetic resonance imaging versus conventional angiography. *Neurol. Med. Chir. (Tokyo)* 51 195–200. 10.2176/nmc.51.195 21441735

[B9] Kamada F. Aoki Y. Narisawa A. Abe Y. Komatsuzaki S. Kikuchi A. (2011). A genome-wide association study identifies RNF213 as the first Moyamoya disease gene. *J. Hum. Genet.* 56 34–40. 10.1038/jhg.2010.132 21048783

[B10] Kamimura T. Okazaki S. Morimoto T. Kobayashi H. Harada K. Tomita T. (2019). Prevalence of RNF213 p.R4810K variant in early-onset stroke with intracranial arterial stenosis. *Stroke* 50 1561–1563. 10.1161/strokeaha.118.024712 31060437

[B11] Koizumi A. Kobayashi H. Hitomi T. Harada K. H. Habu T. Youssefian S. (2016). A new horizon of moyamoya disease and associated health risks explored through RNF213. *Environ. Health Prev. Med.* 21 55–70. 10.1007/s12199-015-0498-7 26662949 PMC4771639

[B12] Koizumi A. Kobayashi H. Liu W. Fujii Y. Senevirathna S. T. M. L. D. Nanayakkara S. (2013). P.R4810K, a polymorphism of RNF213, the susceptibility gene for moyamoya disease, is associated with blood pressure. *Environ. Health Prev. Med.* 18 121–129. 10.1007/s12199-012-0299-1 22878964 PMC3590321

[B13] Komiyama M. (2017a). Cardio-cephalic neural crest syndrome: a novel hypothesis of vascular neurocristopathy. *Interv. Neuroradiol.* 23 572–576. 10.1177/1591019917726093 28814167 PMC5814071

[B14] Komiyama M. (2017b). Moyamoya disease is a vascular form of neurocristopathy: disease of the embryologic cephalic neural crest. *Childs Nerv. Syst.* 33 567–568. 10.1007/s00381-017-3369-2 28299436

[B15] Kondori B. J. Azemati F. Dadseresht S. (2017). Magnetic resonance angiographic study of anatomic variations of the circle of Willis in a population in Tehran. *Arch. Iran. Med.* 20 235–239. 28412828

[B16] Liu W. Morito D. Takashima S. Mineharu Y. Kobayashi H. Hitomi Y. (2011). Identification of RNF213 as a susceptibility gene for moyamoya disease and its possible role in vascular development. *PLoS One* 6:e22542. 10.1371/journal.pone.0022542 21799892 PMC3140517

[B17] Matsuda Y. Mineharu Y. Kimura M. Takagi Y. Kobayashi H. Hitomi T. (2017). RNF213 p.R4810K variant and intracranial arterial stenosis or occlusion in relatives of patients with Moyamoya disease. *J. Stroke Cerebrovasc. Dis.* 26 1841–1847. 10.1016/j.jstrokecerebrovasdis.2017.04.019 28506590

[B18] Merkkola P. Tulla H. Ronkainen A. Soppi V. Oksala A. Koivisto T. (2006). Incomplete circle of Willis and right axillary artery perfusion. *Ann. Thorac. Surg.* 82 74–80. 10.1016/j.athoracsur.2006.02.034 16798193

[B19] Miyawaki S. Imai H. Takayanagi S. Mukasa A. Nakatomi H. Saito N. (2012). Identification of a genetic variant common to moyamoya disease and intracranial major artery stenosis/occlusion. *Stroke* 43 3371–3374. 10.1161/STROKEAHA.112.663864 23010677

[B20] Morito D. Nishikawa K. Hoseki J. Kitamura A. Kotani Y. Kiso K. (2014). Moyamoya disease-associated protein mysterin/RNF213 is a novel AAA+ ATPase, which dynamically changes its oligomeric state. *Sci. Rep.* 4:4442. 10.1038/srep04442 24658080 PMC3963067

[B21] Okazaki S. Morimoto T. Kamatani Y. Kamimura T. Kobayashi H. Harada K. (2019). Moyamoya disease susceptibility variant RNF213 p.R4810K increases the risk of ischemic stroke attributable to large-artery atherosclerosis. *Circulation* 139 295–298. 10.1161/CIRCULATIONAHA.118.038439 30615506

[B22] Research Committee on the Pathology, and Treatment of Spontaneous Occlusion of the Circle of Willis, and Health Labour Sciences Research Grant for Research on Measures for Intractable Diseases (2012). Guidelines for the diagnosis and treatment of Moyamoya disease (spontaneous occlusion of the circle of Willis). *Neurol. Med. Chir. (Tokyo)* 52 245–266. 10.2176/nmc.52.245 22870528

[B23] Roher A. E. Esh C. Kokjohn T. A. Kalback W. Luehrs D. C. Seward J. D. (2003). Circle of Willis Atherosclerosis is a risk factor for sporadic Alzheimer’s disease. *Arterioscler. Thromb. Vasc. Biol.* 23 2055–2062. 10.1161/01.ATV.0000095973.42032.44 14512367

[B24] Song P. Qin J. Yu Y. Shi C. Qiao P. Xie A. (2019). Comparative performance of magnetic resonance angiography and digital subtraction angiography in vessel involvement of pediatric moyamoya disease. *Iran. J. Radiol.* 16:e5559. 10.5812/iranjradiol.55595

[B25] Yoshimoto T. Inoue M. Tanaka K. Kanemaru K. Koge J. Shiozawa M. (2021). Identifying large ischemic core volume ranges in acute stroke that can benefit from mechanical thrombectomy. *J. NeuroInterv. Surg.* 10.1136/neurintsurg-2020-016934 (in press). 33323502 PMC8606466

