# Supplementary material for: RNF213 p.R4810K (c.14429G > A) Variant Determines Anatomical Variations of the Circle of Willis in Cerebrovascular Disease
Source: Front Aging Neurosci. 2021 Jul 15;13:681743. doi: 10.3389/fnagi.2021.681743 (PMC8322682; doi:10.3389/fnagi.2021.681743)
Supplement: Supplementary file 1 [file Table_1.pdf]

Supplementary Material

RNF213 p.R4810K (c.14429G>A) variant determines anatomical variations of the circle of Willis in cerebrovascular disease

Futoshi Eto[1,2†], Takeshi Yoshimoto[1†], Shuhei Okazaki[1,3], Kunihiro Nishimura[4], Shiori Ogura[1, 2], Eriko Yamaguchi[1,2], Kazuki Fukuma[1], Satoshi Saito[1], Kazuo Washida[1], Masatoshi Koga[2], Kazunori Toyoda[2], Takaaki Morimoto[5,6], Hirofumi Maruyama[7], Akio Koizumi[6,8] and Masafumi Ihara[1*] †These authors have contributed equally to this work.

Supplementary Table I. Comparisons of stroke subtypes between large artery atherosclerosis and non-large artery atherosclerosis in the cohort (pages 2–3).

Supplementary Table I. Comparisons of stroke subtypes between large artery atherosclerosis and non-large artery atherosclerosis in the cohort.

	**Large artery atherosclerosis (*n* = 139)**	**Small-vessel occlusion (*n* = 135)**	**Other/undetermined etiology (*n* = 185)**	*P*-value
**Women, *n* (%)**	32 (23)	34 (25)	71 (38)	<0.01
**Age, median (IQR), years**	73 (61–79)	70 (63–78)	64 (50–78)	<0.01
**Prestroke mRS score, median (IQR)**	0 (0–0)	0 (0–0)	0 (0–0)	0.82
**Baseline systolic BP, median (IQR), mmHg**	162 (140–175)	162 (144–177)	162 (143–179)	0.26
**Medical history**				
**Hypertension, *n* (%)**	122 (88)	114 (84)	131 (71)	<0.01
**Diabetes mellitus, *n* (%)**	52 (37)	44 (33)	34 (18)	<0.01
**Dyslipidemia, *n* (%)**	111 (80)	86 (64)	112 (61)	<0.01
**Atrial fibrillation, *n* (%)**	2 (1.4)	1 (0.7)	3 (1.6)	0.88
**Current smoking, *n* (%)**	96 (69)	85 (63)	81 (44)	<0.01
**Ischemic heart disease, *n* (%)**	12 (9)	7 (5)	7 (4)	0.17
**Chronic kidney disease, *n* (%)**	52 (37.4)	46 (34.1)	47 (25.4)	0.05
**Baseline NIHSS score, median (IQR)**	8 (6–10)	2 (1–4)	5 (2–5)	0.83
**ASPECTS on DWI or CT, median (IQR) (*n* = 465)**	10 (10–10)	10.00 (10–10)	10 (9–10)	0.17
**Formation of the vessels constituting the circle of Willis**				
**Both intracranial ICAs, *n* (%)**	131 (94)	132 (98)	182 (98)	0.11
**Both A1 segments of ACAs, *n* (%)**	121 (87)	120 (89)	162 (88)	0.92
**AComA, *n* (%)**	123 (89)	124 (92)	146 (79)	<0.01
**Top of BA, *n* (%)**	137 (99)	135 (100)	185 (100)	0.18
**Both P1 segments of PCAs, *n* (%)**	104 (75)	108 (80)	149 (81)	0.42
**Both PComAs, *n* (%)**	16 (12)	18 (13)	39 (21)	0.06
**Focal narrowing of the M1 segment of MCA, *n* (%)**	35 (25)	4 (3)	24 (13)	<0.01
**Complete circle of Willis, *n* (%)**	8 (6)	14 (10)	25 (14)	0.07
**mRS score at discharge, median (IQR)**	2 (1–3)	2 (1–3)	1 (1–3)	0.35

**In-hospital mortality, *n* (%)**	0	0	2 (1)	0.34
***RNF213* p.R4810K (c.14429G>A) variant carriers, *n* (%)**	14 (10)	5 (4)	4 (2)	<0.01

Abbreviations: ACAs, anterior cerebral arteries; AComA, anterior communicating artery; ASPECTS, Alberta Stroke Program Early CT Score; BA, basilar artery; BP, blood pressure; CT, computed tomography; DWI, diffusion-weighted imaging; ICAs, internal carotid arteries; IQR, interquartile range; MCA, middle cerebral artery; mRS, modified Rankin Scale; NIHSS, National Institutes of Health Stroke Scale; PCAs, posterior cerebral arteries; PComAs, posterior communicating arteries.
